# Radiotherapy-induced localized bullous pemphigoid with a favorable response to dupilumab

**DOI:** 10.1016/j.jdcr.2024.04.013

**Published:** 2024-04-21

**Authors:** Chanidapa Wongtada, Pawinee Rerknimitr

**Affiliations:** Division of Dermatology, Department of Medicine, Faculty of Medicine, Center of Excellence for Skin and Allergy Research (CESAR), Chulalongkorn University, Bangkok, Thailand

**Keywords:** autoimmune bullous disease, bullous pemphigoid, dupilumab, radiotherapy

## Introduction

Bullous pemphigoid (BP) is an autoimmune blistering disease characterized by the presence of autoantibodies to hemidesmosome protein BP antigen 1 (BP230) and 2 (BP180). Typical presentation includes itchy and widespread tense blisters on apparently normal or erythematous skin; however, several variants have been described. Localized BP, a variant with lesions restricted to a body region, is often associated with local trigger factors such as radiation, phototherapy, medications, trauma, and surgery.^1^ In this report, we present a case of radiotherapy (RT)-induced localized BP who achieved a promising outcome following dupilumab administration.

## Case report

**Fig 1:**
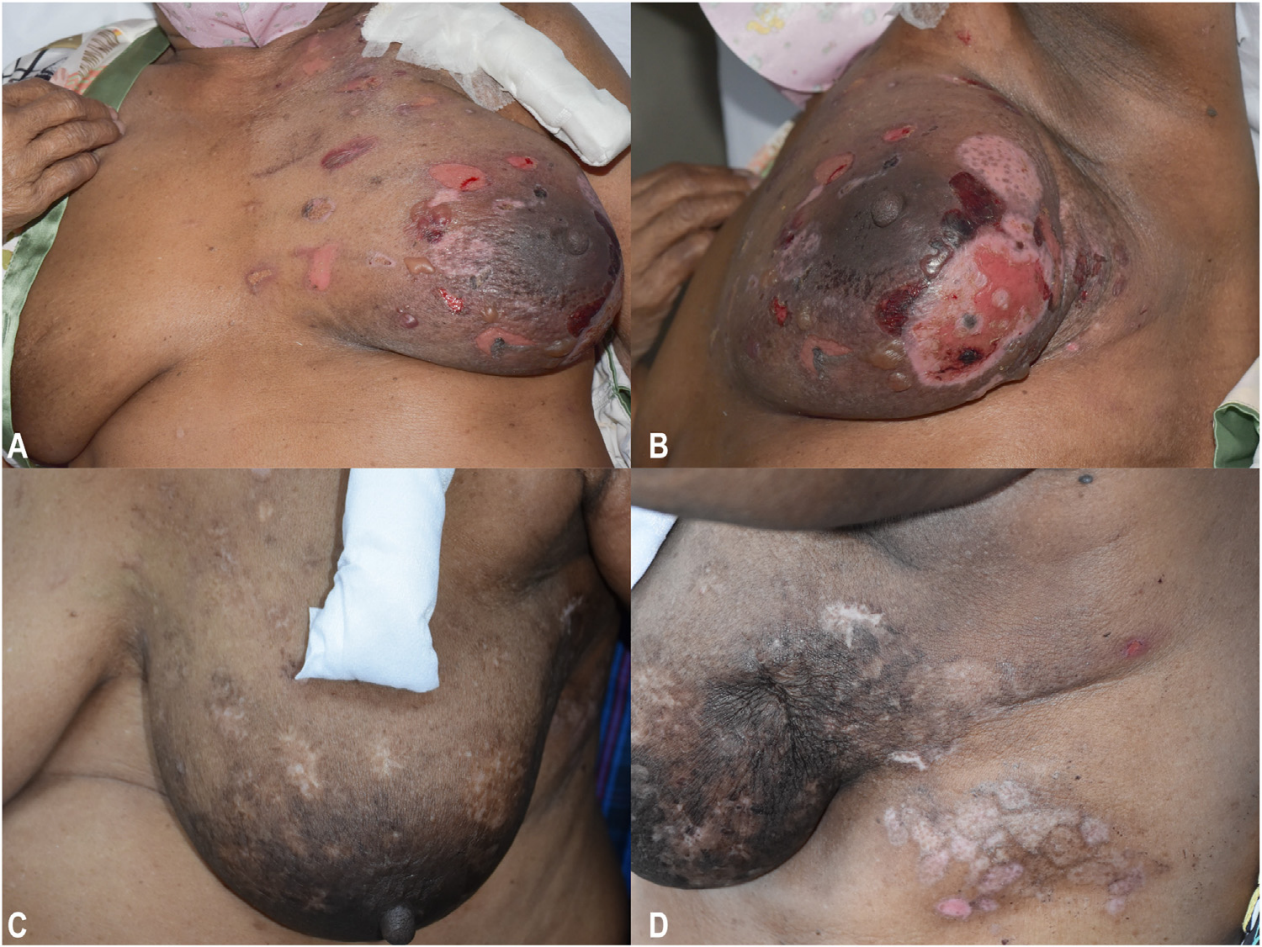
Radiation-induced bullous pemphigoid. **A, B,** Multiple serous and hemorrhagic tense bullae and erosions confined to the left breast (before treatment). **C, D,** Improvement of bullae and erosions post dupilumab therapy.

**Fig 2:**
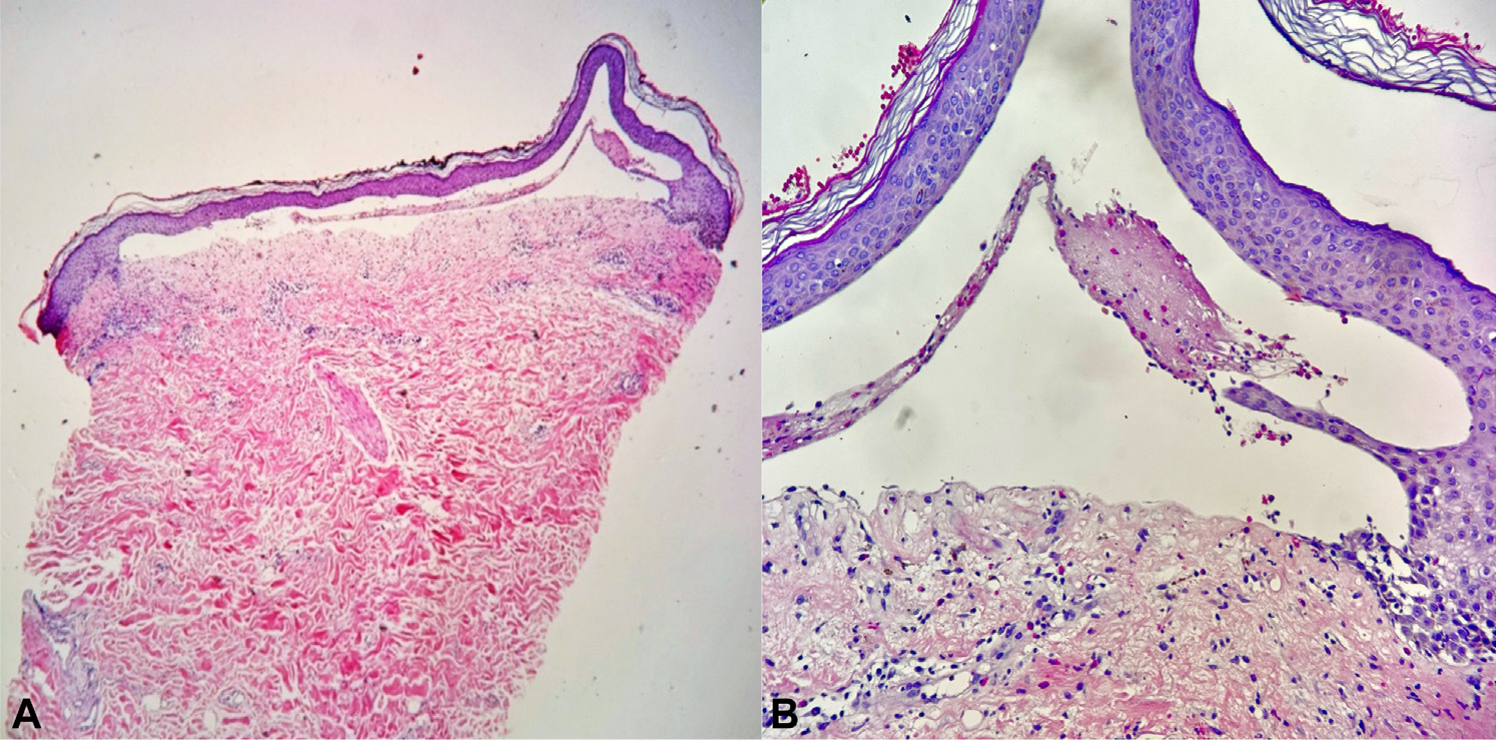
Histopathologic examination shows subepidermal separation with superficial perivascular and interstitial mixed cell infiltrate containing numerous eosinophils. (Hematoxylin-eosin stain; original magnifications: **A,** ×40; **B,** ×400.)

A 79-year-old woman with underlying end-stage kidney disease, ischemic heart disease, atrial fibrillation, and hypertension was diagnosed with left breast invasive mammary carcinoma, grade 3, human epidermal growth factor receptor 2 positive, stage T2N1M0 in 2022. After tumor resection, she received trastuzumab as an adjuvant targeted therapy and underwent RT of 26 Gy in 5 fractions on her left breast and regional lymph nodes for 2 weeks. One month following RT, she reported development of blisters mainly localized on her left breast, which later became eroded (Fig 1, *A, B*). Mucosal involvement was absent. Histopathologic examination revealed a subepidermal blister with superficial perivascular and interstitial mixed cell infiltrate containing numerous eosinophils (Fig 2). Direct immunofluorescence study showed no deposition of immunoreactants; however, the enzyme-linked immunosorbent assay for BP antigen 1 and 2 antibodies were positive at >200 and 138.7 RU/mL (normal value, <20 RU/mL), respectively. Based on these findings, the diagnosis was consistent with RT-induced localized BP since the blisters were only confined to the area with RT exposure. Oral prednisolone 30 mg/d was initially prescribed for 3 weeks, followed by topical treatment only with clobetasol propionate cream for 3 months. However, flares of blisters and erosions were still ongoing. Following discussion on treatment choices with the patient and taking her comorbidities into account, subcutaneous injection of dupilumab 300 mg was implemented along with topical corticosteroid. Three weeks later, the lesions resolved (Fig 1, *C, D*) and the patient remained bullous-free for 3 months following a single dose of dupilumab. However, recurrence prompted the administration of dupilumab at 4- to 6-week intervals due to the patient’s financial constraints. No adverse events were reported.

## Discussion

Since the initial documentation of RT-induced BP in 1988, several studies have confirmed a connection between RT and BP.^2^, ^3^, ^4^ Other factors such as breast cancer; chronic kidney disease, as seen in our case; and concurrent chemotherapy have also been regarded as significant risk factors for causing RT-induced BP.^2^ Although the underlying mechanisms are not fully understood, several hypotheses have been proposed. One of the hypotheses suggests that RT may locally alter the antigenic properties of the basement membrane proteins and unmask them. Exposing antigens to CD4^+^ T cells prompts the development of autoantibodies that subsequently bind BP antigens and trigger complement activation and leukocyte attraction, resulting in the cleavage of the lamina lucida and bullae formation.^1^, ^2^, ^3^, ^4^ Another hypothesis suggests that RT may enhance the deposition of preexisting circulating antibodies on the basement membrane of the tissue injured from RT, probably through an alteration of permeability of blood vessels.^1^, ^2^, ^3^ RT could also cause immune dysregulation and inhibition of T-cell suppressor activity, contributing to an unopposed T-cell helper function and overproduction of basement membrane antibodies.^1^^,^^4^

Treatment approaches are similar to idiopathic BP, including topical corticosteroid and systemic treatment using prednisolone or immunosuppressive agents.^1^^,^^4^ A combination of tetracycline and niacinamide or anti-CD20 agents for refractory cases have also been employed.^1^ Patients with RT-associated BP mostly experience a favorable clinical outcome.^3^ Nonsystemic corticosteroid treatments are usually sufficient to control the disease,^1^ although some patients may eventually require systemic treatment.^3^^,^^4^ Based on previous reports, BP developed in most patients after completing RT rather than during RT sessions.^3^ Among those who experience BP during RT, there is no consensus regarding RT discontinuation.^3^^,^^5^^,^^6^ In fact, it is considered case by case weighing risks and benefits of RT. If the benefit still outweighs the risk, RT interruption may not be necessary and parallel treatment of BP should be implemented. However, whether continuing RT could alter the course of RT-induced BP or not still needs further studies.

In recent years, dupilumab has been considered an alternative treatment for BP, especially in recalcitrant BP or in older patients with comorbidities who are at risks of adverse events from systemic corticosteroid or immunosuppressive agents.^7^ Dupilumab is a fully human IgG4 monoclonal antibody targeting interleukin 4 receptor α, which results in the reduction of interleukin 4 and interleukin 13 signaling in T helper 2–type responses.^8^^,^^9^ Since these cytokines are involved in the pathogenesis of BP and participate in eosinophil recruitment and anti-BP180 antibody production, dupilumab is considered a promising treatment for BP.^7^^,^^9^ A number of publications have documented the efficacy of dupilumab, as an adjunctive therapy or monotherapy, in treating BP.^7^^,^^10^ A recent multicenter retrospective cohort study demonstrated that dupilumab was associated with rapid and sustained improvement of clinical symptoms and serologic indicators in BP with a favorable safety profile.^9^ The dosage used for adult patients with BP in the studies was 300 mg of dupilumab every 2 weeks, following an initial dose of 600 mg. Additionally, drug-induced BP was excluded from the study.^9^ Dupilumab was also shown to possess a similar efficacy to systemic corticosteroid in treating BP.^8^ In our case, despite the patient’s inability to adhere to the recommended dosage due to financial concerns, she responded positively to the minimal dose/interval of dupilumab without concurrent systemic steroid use. The patient has been followed up for 5 months with continued administration of dupilumab.

Until now, there have been numerous reports of RT-induced BP treated with conventional therapy; however, data on cases treated with dupilumab are still limited. This case not only underlines the role of RT in triggering BP but also showed a satisfactory outcome after dupilumab treatment. Along with its well-tolerated profile, dupilumab could be worth considering in patients with RT-induced BP as such cases typically occur among patients with cancer who are elderly with multiple comorbidities or in immunocompromised state.

## Conflicts of interest

None disclosed.
